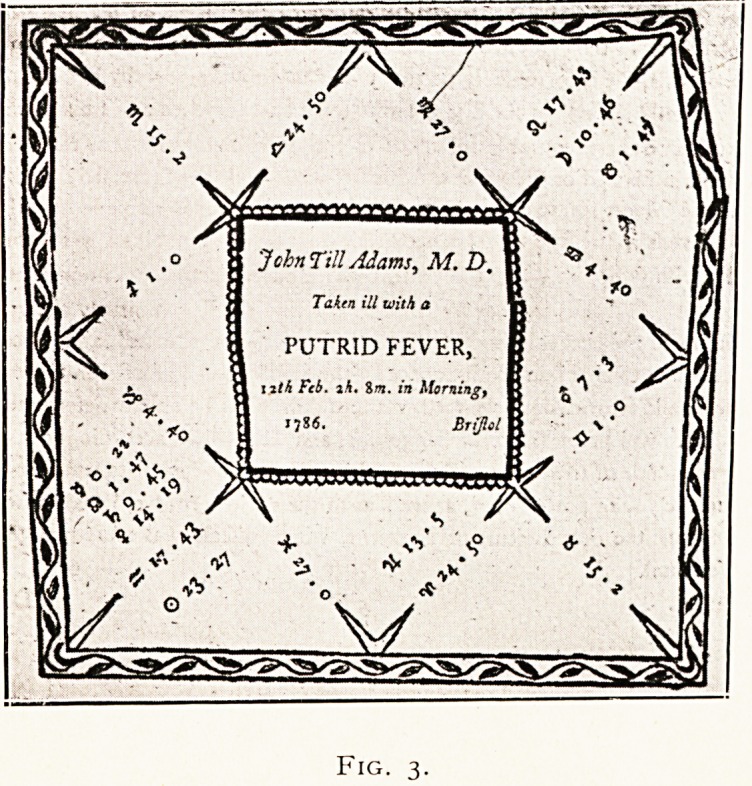# Contemporary Medicine from the Standpoint of Pathology

**Published:** 1919-12

**Authors:** I. Walker Hall

**Affiliations:** Professor of Pathology, Bristol University


FIG. 1.

FIG. 2.

FIG. 3.

### The Bristol Medico-Chirurgical Journal.

*"Scire est nescire, nisi id me
Scire alius sciret."*

DECEMBER, 1919.

Library of Congress
533694
MAR 19 1920
Smithsonian Deposit

### CONTEMPORARY MEDICINE FROM THE STANDPOINT OF PATHOLOGY.

The Presidential Address, delivered on October 8th, 1919, at the opening of the Forty-sixth Session of the Bristol Medico-Chirurgical Society.

BY

I. WALKER HALL, M.D.,

*Professor of Pathology, Bristol University.*

It is a pleasant duty to express my appreciation of the honour conferred by you in permitting me to occupy the Presidential Chair of this well-known and highly-esteemed Society. A close association with its work during the last thirteen years has rewarded me with many happy memories. Foremost among these I place the friendships of those former Presidents and Officers whose lamps went out while the war was raging. To Munro Smith, Mole, Michell Clarke, and others we owed much, while the influences they exerted at our meetings will remain with many of us until we too reach the Gates of the West.

Perhaps I may be pardoned if I venture to hope that the selection of a representative of the subject of pathology as your President will not divert your clinical interests unduly. It may be that your action presages a more binding union between the laboratory and the bedside and a more intimate understanding of the objects of each. At all events, I have been led to think that a review of these relations in the light of history, experience and outlook may not be without its interest and stimulus.

Such a consideration of contemporary medicine from the standpoint of pathology enables me at once to revert to a period when each practitioner undertook entirely his own medical work. It permits me to scan the well-known developments of medical sciences, not from the accounts of the great pioneers themselves, but through the spectacles of those men whose practice was influenced by the various discoveries which lessened suffering and prevented disease.

Turning first to the English edition of the *General Practice of Physicke,* by Christopher Wirtzung, published by Thomas Adams, of London, in 1617, we are told (p. 3) :—

"That the reason of man seemeth to complain greatly, that our nature should be subject unto so many infirmities, miseries and calamities ; Yea, it doth as it were, chide with God, and accuse him of tyranny, for creating man so wretched. And, in very deed, a worldly minded man, that neither acknowledgeth God, nor the *cause* of his natural imperfections, might easily be brought and carried away with such like opinions, and with great reason bewail the infinite wretchedness of mankind, seeing he findeth no creature on the face of the whole earth that is vexed with so many infirmities, but man only : But contrariwise, they that are endued with the knowledge of God and godliness, will not accuse him of tyranny that is most gentle ; will not accuse him of evil, that hath created and made all things most perfect and good, nay, that never could nor would do harm. Adam was created in the beginning, sound and whole, perfect, wise, absolute and without blemish or spot whatever: but by disobedience, transgressing the commandment of God, in that he would, without contradiction, be made like unto God, and know both good and evil, which he did of his own stubborn head, and in despite and contempt of his Creator: herewith hath he not only brought and wrought unto himself, but unto all his posterity after him, an everlasting mishap and infelicity, as a well worthy and deserved punishment for his and our misdeeds together. Wherefore sin is the first and only cause of all our distress, of all our sicknesses and heaviness here on earth: which has brought us to that state, that after all our turmoiling and painstaking, we must at the last taste and take death as a merit and payment for our sins."

It was not to be expected that this finality of causation would gain prolonged acceptance in a period when philosophers generally were pushing back the causal factors of existence by argument and experiment, and Bacon was, at the moment, illuminating thought with his methods of analysis.

Hence, we are not surprised to find that thirty years after the death of Bacon, Riverius in his book on *General Medicine*, published by Henry Everden at the Greyhound in St. Paul's Churchyard in 1657, tells us (p. 116):—

"A physician ought always in the legitimate and rational cure of preternatural affections, to observe this method: First, he must act the industrious scrutinist, in enquiring after the nature and causes of the proposed disease and the situation of the part affected. Secondly, he must make a diligent search, whether the cause be not desperate and the disease incurable: that hence we may gather, that remedies fit for the acquisition of health are to be applied, if our Art upon serious inspection promise a recovery: or if the prevalent disease delude our skill to a despair: forthwith to abstain from administering anything, lest those applications which have procured health to any, should be undervalued to a dis-esteem."

This recognition that the old Adam is not the only cause of disease is a belated tribute to the influence of Bacon and other philosophers; but it is a quaint mind which would preserve faith in tried remedies by reserving them for cases with a good prognosis.

It reminds one of Thomas Carlyle, who once remarked that "had Adam remained in Paradise, there had been no anatomy and no metaphysics," and went on to contend that "the beginning of enquiry is disease; all science, if we consider well, must have originated in the feeling of something being wrong." "Thus," continued the old satirist, "the Tree of Knowledge springs from a root of evil, and bears fruits of good and evil." [1]

Riverius defines disease as "a disposition of a body preternatural, primarily and by itself injuring the actions." "If we deliberate of action itself," he goes on to say, "if it be hurt in man's body, we say it is sick; but if whole and unhurt, we say it is in health. I call that action hurt, which manifestly and sensibly appeareth such. Moreover, action being a motion, and having no permanent essence, only so long in being as it is doing and performing; therefore it implies the necessity of a constant and permanent cause. If therefore the body be in health, when being naturally disposed, it produceth perfect actions; it will be diseased, when it being disposed beside nature, it exerts actions imperfect, and so this detriment of actions will depend upon this preternatural disposition."

There is much in this that accords with current ideas upon re-action to irritants; it seems as if the same fundamental question agitated human minds century after century.

Riverius considers in detail the nature of morbid causes, and concludes that in disease the efficient is the only effective one.

"The philosopher rallies all the causes of things under four heads, viz., Formal, Material, Efficient and Final." The efficient is the only considerable one. It is not only that from which the effect is first produced, but stands for all that which is in any manner conducible to the generation of the disease.

The critical days of a disease form a subject which looms large in the mind of our author. He defines crisis as a "kind of compound, comprehending conturbation, evacuation and sudden mutation to health." Conturbation is a plenty of critical symptoms arising from the agitation of morbid matter. The principal cause of this agitation is the motion of the moon, which moves the humors every critical day and excites nature to evacuation. But there must be other causes of an internal nature, for nature does not attempt exertion, unless she finds matter prepared and disposed for evacuation. Still, the crisis, or critical evacuation, which happens in the septenary or quatenary days, is due to the virtue of the moon.

Were I to detail the many subsidiary causes which Riverius marshals with striking confidence, it would be evident to you that he is trying to break away from old traditions even while he feels himself shackled by current thought. But before I examine the persistence of supernatural influences, you will perhaps permit me to refer to a few rhymes which Riverius ascribes to the Salerno School that was so dominant from 1300 to 1500 A.D. It is very curious how odd phrases and rhymes have crystallised certain ideas, which otherwise would have had their day and ceased to be :—

> " At supper if you fill with meat
> Your stomach, your pain will be great ;
> If you desire a quiet night,
> Make a supper short and light."
>
> " After supper stand a while,
> Or else walk a mile."
>
> " Beware how thou eatest crust,
> For it produces choler adust."
>
> " Beets nourish little, I must tell ye,
> They do both bind and loose the belly."
>
> " Of cabbage this for certain we do find
> The broth doth loosen, but the substance bind."

And now we come to a period when from our charming Wrington village John Locke went on to Oxford to apply his genius to medical research and to gain such high approbation from Sydenham ; when Sir Thomas Browne foresaw the growth of tropical medicine, writing in one of his letters, " New discoveries of the earth discover new diseases ; for besides the common swarm, there are endemial and local infirmities proper unto certain regions which in the whole earth make no small number ; and if Asia, Africa and America should bring in their list, Pandora's box would swell and there must be a strange pathology " ;[2] and when Morgagni was in the hey-day of his startling work on morbid changes. What was the effect of all this on contemporary medicine ?

I turn to a volume entitled a *Key to Physic and the Occult Sciences*, published some twenty-seven years after the death of Morgagni, by E. Sibley, as a supplement to his edition of Culpeper's *English Physician.*

His frontispiece (Fig. 1) is a strange mixture of alchemy, geometry, astronomy and mysticism. Those acquainted with the symbolism of secret societies will see much in it of interest. He dismisses the purely pathological aspect of man in the following words: "*Memento mori!* The life of man resembles a bubble ready to burst; his fate is suspended by a hair, and is dependent upon the uncertain lapse of time." "Life," he considers, "begins in motion and ends in rest." Disease is a "perturbation in the progressions of motion," a new phrase for action hurt.

He upholds strongly the doctrine of expulsive force, or evacuation of foul and offending humors from the blood by means of insensible perspiration and provides a special plate for its demonstration (Fig. 2), remarking that "it passes through our garments with the utmost ease, particularly, if woollen; and it even ascends through the bedclothes like a mist, in the greatest abundance when we are asleep, and the animal functions are at rest."

But his main thesis is the action of the sun and planets upon the internal organs. His predominant idea is prognosis with celestial influences as his guide. He gives a tangible instance in the decumbiture of Dr. John Till Adams, of Bristol (Fig. 3). This Adams, according to Munro Smith, assisted Borlase in his difficulties with the Infirmary staff. He practised chiefly among Quakers, and his death opened the way for the coming of Long Fox. Sibley himself also worked in Bristol, for he states: "These figures I erected while resident in Bristol, at the request of my good friend, Dr. Till Adams." From an exposition of the decumbiture he derived the factors which "put an end to the existence of this much respected man. Eight days after he was seized by a fever and six days after it was foretold by the horoscope; from whence, having foreseen the doctor's fate, I composed an Elegy on his death, while he was yet alive, which I got printed and published the very day he expired, thus manifesting to the world, with the patient's earnest approbation, an incontrovertible instance of the verity of astral prediction."

A similarly accurate forecast was made concerning the French Revolution and the violent deaths of the King and Queen six years before they occurred.

Yet this good man, confident in his beliefs, relied much upon a certain solar tincture be obtained by re-distillation of vegetable residues, and includes in his work the following letter :—

*To* E. SIBLEY, M.D.

SIR,

In gratitude, I cannot but thank you for that excellent medicine, the solar tincture. It has saved my life. I was suddenly seized with a violent cholic, which brought on mortification of the bowels. The efforts of the Faculty were tried in vain, and I was given over. In these moments of extremity, my existence was preserved by only two spoonfuls of your medicine, undiluted, which instantly relieved me from the wrack of torture. After two more doses, the obstruction was removed by natural evacuation, and a few hours restored me to my usual state of good health. I entreat you to publish this for the public good and shall be ever,

Gratefully yours,

JOHN POWELL.

CLIFTON, NEAR BRISTOL,
*Feb.* 24, 1794.

It is worthy of mention that four years before Sibley issued his treatise, the notebook of a nameless medical student contained a record of the post-mortem findings upon a wet nurse, E. C., who died at the Bristol Royal Infirmary on March 7th, 1794. The report is carefully transcribed and the pulmonary morbid changes are clearly stated ; while it indicates that teaching in pathological anatomy was already a feature of the day.

This was an observant student, for his notes include the following :—

"It has been disputed whether the menstrual blood comes from the vagina or the uterus. A case lately occurred where there was so great a prolapsus uteri that the Os Tincæ hung a considerable way below the labia pudendi, and at the time of Menstruation the Blood was found to ooze out from the internal part of the Os Tincæ, which proves that the Blood comes from the uterus."

Some forty years later Richard Smith gave an address at the opening of the Bristol Medical School at Old Park (October 14th, 1834), in which he summarised clearly the attitude of current medical thought towards scientific developments. He said : "The origin and nature of those changes of composition and texture to which every organ is liable and which form the prelude to those changes of structure, first affecting functions and then altogether destroying them, are the subjects comprehended by morbid anatomy. This substitutes demonstration for theory and conjecture, and lays the solid foundation for rational practice. By the study of morbid anatomy the student will soon lose the feelings of disgust at the sight of diseases even the most hideous. His feeling is changed for one of a more abstract character ; he sees in the case only peculiar conditions of the cellular tissues ; increased action of the absorbents ; increased vascularity ; turgescence of the veins or vitiated secretions of the capillaries."

This bespeaks a breadth of vision hardly expected eight years before Schwann outlined his cellular theory of the organism and fourteen years before Virchow stated his views upon cellular pathology. Had it become more general among the clinicians, the limitations of morbid anatomy would have been recognised at an earlier date, and the end stages of disease would have less often formed the mental picture which the student carried to the bedside during the early days of an illness. But the gross structural changes held the attention for long; the abstract visualisation was a capacity as rarely apparent as it is hard to stimulate to-day.

The examination of the morbid changes in tissues demanded more time, however, than the clinician could bestow. The pathologist was born. For long he was the servitor of the physicians and surgeons. Perhaps in making him the Cinderella of the art the elder sisters forgot the ending of the story.

So, for forty years more medicine clung to the *Sectio* and the sections. Men had forgotten that striking experiment of Bacon which practically caused his death. How the old man was driving in his carriage on a snowy day, when he conceived the idea that cold might prevent putrefaction. He stopped at a cottage, procured a fowl, had it killed, stuffed it with snow and confirmed his thought. The clinician of the period seemed also to be unaware of the fundamental and exhaustive work of Bossi—published but twelve months after Richard Smith had given his address—which laid the foundation of subsequent bacteriological developments. Until Pasteur and Koch in 1877 startled the medical world by the applications of Bossi's technics, contemporary medicine had lagged behind. Much of their work is but now filtering slowly into general practice, although pathology has since then extended new branches of morbid physiology, hæmatology, protozoology, pathological chemistry, serology, immunology, etc.

What is the position of contemporary medicine to-day in its relation to current pathology? The text books are numerous. In all, or nearly all, the contained pathology is the current teaching of the hour. If the seventeenth and eighteenth centuries required fifty years for the incorporation of new conceptions and the late nineteenth called for a decade, the twentieth century presents its clinicians with yearly or half-yearly editions brought up to date of publication. But pathology, in common with other subjects, has developed its own terminology ; in my haste, I had almost written its own Babel. Hence, although the matter is up to date, its intelligent appreciation is precluded until the reader is *au fait* with the occult. The epopt is excluded from the elect.

So it is that although we derive our status from a faithful stewardship of the " knowledge which has been garnered for us by an illustrious brotherhood of men—many of whom paid for it by their lives," we are split up into sections, perhaps factions, which tend to preclude any broad conceptions of our trust. Our views are narrowed, our outlook is limited by the very depth of the ruts we have made. We have gone too far apart in our pursuit of the sectional. While we may follow Sir William Gull in his claim that " from the ranks of our profession the chief cultivators of modern science have sprung ; whether we speak of botany, comparative anatomy, chemistry, physiology, biology, hygiene, or social science," we are tempted to forget the converse, namely, that the common ground of these outgrowths is human life, its experience and its possibilities. This fundament may ever be to us an incentive to use all means of intercourse to bring the subdivisions of medical science into correlation. The future calls for a closer link between investigation and practice. The entrusting of the teaching of anatomy, physiology, pathology and special subjects to experts is of benefit only so long as the specialist retains his sense of clinical requirements. The delicacy of this sense depends naturally upon the opportunities for renewal and stimulation ; so that the maintenance of true medical insight calls for a continuous association between the laboratory and the bedside.

Contemporary medicine, on the other hand, may gain much by a more intimate association with the workers in special subjects. "The pathologist," says Gull, "follows the steps of the physiologist, and often their work is but one." Again, "Where hygiene fails, properly commences the work of therapeutics; but it is painful to find ourselves occupied in making feeble, and often useless, efforts to combat the effect of a poison which might perhaps have been stamped out in its beginnings."

Were I a clinician—was that a whisper, "The gods forefend"?—I would not rest content until the staffs of hospitals included anatomists, physiologists, bio-chemists, as well as pathologists, until I could meet them in daily consultation and co-operation in my wards. And I would strain my interest to bring them into working association in out-patient departments, dispensaries, urban and rural clinics. I would urge that in the building of these institutions there should be suites of rooms radiating outwards from a central nucleus of scientific laboratories. A pathologist should be my colleague in the examination of all new, and apparently trivial, cases. With him I would undertake all possible, and seemingly impossible, examinations upon patients exhibiting the beginnings of dyspepsia, headache, catarrhs, amenorrhœa, sexual mentalities, and anomalies of metabolism. The anatomist and physiologist should be my mentors in the slighter ailments of childhood, and of puberty, etc. On certain days the practitioners should join me and enlighten my staff with their experiences, inquiries, and difficulties. The industrial hygienist should have his place also, and bring for observation and discovery the early symptoms of altered function or lowered resistance.

I am convinced fully that in such a working co-operation there would be great economies in time and material, while the general results would comprise a more rapid advance in the prevention of disease. I dare to assert that a decade of such conjoined medical effort would bring immeasurable advantage to the health of the community and the maintenance of our industrial units. I cannot visualise any sounder investment, or one presenting greater possibilities of capital appreciation, than that which is wisely directed towards the control of the early stages of disease.

My feelings of gratitude for what Bristol has already done, and my personal obligations to you as a Society, are intensified when I remember that Locke wrote—

> " Nothing in this world is single.
> All things by a law divine
> In another's being mingle."

While I learn anew the lesson of humility, as Kingsley sings to me—

> " Each has his gift—
> Our souls are organ pipes of diverse stop
> And various pitch ; each with its proper notes
> Thrilling beneath the self same breath of God,
> Though poor alone, yet joined, they 're harmony."

REFERENCES.

[1] *Characteristics.* 1831. Thomas Carlyle. } Quoted by E. Blyth. *Ways of Healing.* Heffer, Cambridge, 1919.

[2] *Letter to a Friend.* Sir Thomas Browne } Quoted by E. Blyth. *Ways of Healing.* Heffer, Cambridge, 1919.

## Figures and Tables

**Fig. 1. f1:**
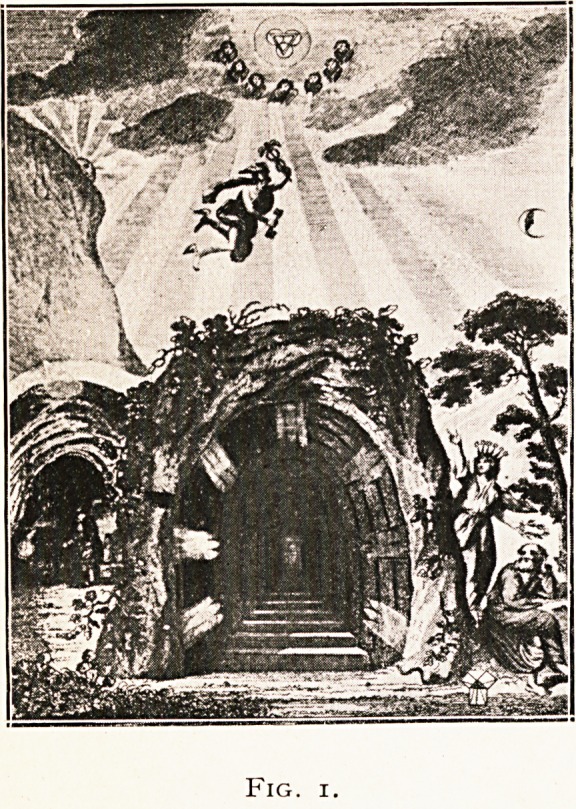


**Fig. 2. f2:**
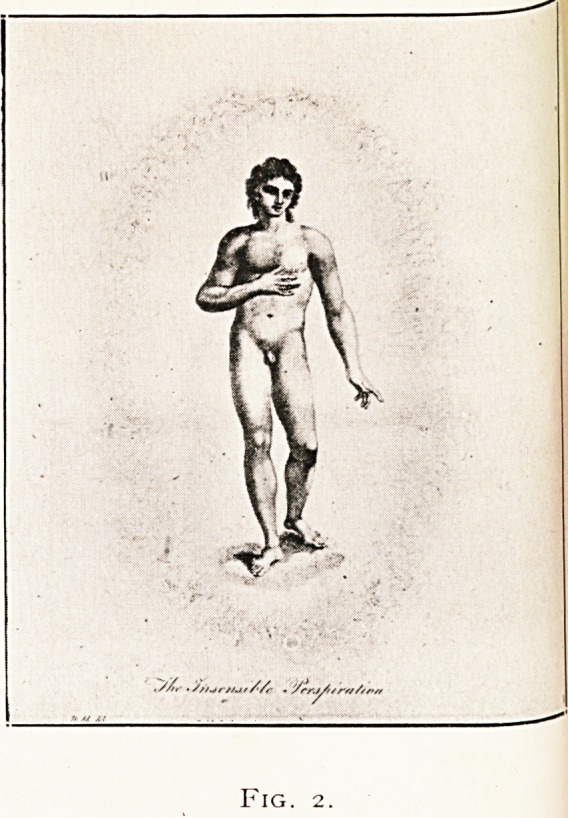


**Fig. 3. f3:**